# Monitoring Lead–Phosphorus Interactions Through ^31^P-NMR Used as a Sensor in Phosphine Functionalized Silica Gel Adsorbent

**DOI:** 10.3390/gels11080580

**Published:** 2025-07-26

**Authors:** Jessica Badillo-Camacho, José A. Gutiérrez-Ortega, Ilya G. Shenderovich, Yenni G. Velázquez-Galván, Ricardo Manríquez-González

**Affiliations:** 1 Department of Wood, Cellulose and Paper, University of Guadalajara-CUCEI, Km 15.5 of Carretera Guadalajara-Nogales, Zapopan 45020, Jalisco, Mexico; jessica.bcamacho@academicos.udg.mx; 2 Department of Chemistry, Universidad de Guadalajara-CUCEI, Blvd. Marcelino García Barragán #1421, Guadalajara 44430, Jalisco, Mexico; 3 Faculty of Chemistry and Pharmacy, University of Regensburg, Universitaetstrasse 31, 93053 Regensburg, Germany; ilya.shenderovich@chemie.uni-regensburg.de; 4 Department of Mathematics and Physics, Instituto Tecnológico y de Estudios Superiores de Occidente (ITESO), Periférico Sur Manuel Gómez Morín 8585, Tlaquepaque 45604, Jalisco, Mexico; yenni.velazquez@iteso.mx

**Keywords:** sol–gel, functionalized silica gel, phosphine, lead adsorption, lead–phosphorus interaction, solid-state NMR

## Abstract

A triphenylphosphine-functionalized silica gel material, optimized for lead adsorption, was synthesized via a one-pot sol–gel reaction and characterized using FTIR and solid-state ^13^C and ^29^Si NMR and XPS spectroscopy. The interaction between lead cations and phosphine groups was evaluated using the ^31^P NMR chemical shift tensor as a sensor. Two distinct types of phosphine groups, exhibiting different rotational mobility behaviors, were identified, with their ratio influenced by the presence of lead cations. These results suggest that the adsorption behavior of lead on this functionalized silica gel adsorbent can be directly evaluated by its lead–phosphorus interaction. This association was corroborated by the shifting of the binding energies of phosphorus functional groups after lead uptake in the XPS analysis.

## 1. Introduction

Heavy metal ions from industrial discharges into natural water systems have become a global environmental concern [1]. Their presence in the water bodies from different industrial processes clearly affects short- and long-term human health due to their proven toxicity and lack of biodegradability. From this group, arsenic, copper, mercury, lead, nickel, cadmium, and zinc are the most toxic to humans [2,3]. Lead (Pb^2+^) is especially hazardous due to its high toxicity, even at trace concentrations, since this metal ion can be bioaccumulated by chronic exposure [4]. In fact, one of the potential sources of lead exposure in drinking water, not exclusively from industrial wastewater, is old lead-based household water supply infrastructure. The World Health Organization (WHO) determined the lead concentration for drinking water to be 10 µg/L [5]. Several methods have been proposed for the treatment of water polluted with lead and some other heavy metals, including ion exchange, precipitation, flocculation, adsorbent zeolites, activated carbon, functionalized mesoporous materials, metallic oxides, membranes, composites, and biobased materials with chitosan, cellulose, and agriculture or forest crops, among others [6,7,8,9].

One of the most efficient methods for removing heavy metal ions from contaminated water is adsorption using functionalized materials [10,11]. Addressing this challenge, hybrid materials synthesized via the sol–gel process have emerged as highly promising candidates. Their tunable porosity, large surface area, and customizable functionalization make them ideal platforms for incorporating specific binding sites [10,12]. In this regard, several attempts using silica gel hybrid materials for the uptake of lead ions from aqueous solutions have been proposed with outstanding performance and efficiency [9,13,14,15,16]. However, only a few works are committed to exploring a critical step in the development of advanced adsorbents by achieving a molecular-level understanding of the adsorption mechanisms by the direct evaluation of the interactions [17,18,19,20]. On the other hand, solid-state NMR can be applied for the study of noncovalent interactions by the estimation of the chemical shielding anisotropy (CSA). Shenderovich (2013) studied the effect of the phosphorus moieties and lead (II) interactions by ^31^P NMR chemical shift tensor (CST) of phosphine oxides, phosphinic, phosphonic, and phosphoric acids using density functional theory-gauge including invariant atomic orbitals (DFT-GIAO) approach [21]. This method, based on the change in ^31^P NMR chemical shift of the phosphorus signal due to the metal association, was successfully used as the P--Pb interaction sensor in the adsorbent functionalized with methyl phosphonate functional groups onto a silica gel matrix [22]. These results demonstrated that phosphorus-based active sites both exhibit excellent adsorption properties and act as molecular sensors, sensitive to interactions with metal ions.

This study focuses on the direct inspection of the molecular interaction between ligands and heavy metal ions in functionalized silica gel materials. Thus, a new hybrid material, silica gel-aminopropyl tris (4-chlorophenyl)phosphine (SG-APT4PP), was synthesized, which features phosphine groups attached to a silica gel matrix through aminopropyl silane. The material was characterized using BET analysis, FTIR, ^13^C, ^29^Si, ^31^P NMR, and XPS spectroscopy. The functional groups were specifically designed to interact with lead ions and act as molecular sensors of their local chemical environment. To investigate this, ^31^P NMR measurements were conducted on the functionalized material before and after exposure to lead. Changes in the ^31^P chemical shift tensor provide molecular-level insights into the binding mechanisms involved. Furthermore, this lead–phosphorus interaction in the hybrid material was also corroborated by XPS analysis.

## 2. Results and Discussion

### 2.1. Lead Adsorption Test

The maximum lead adsorption capacity of SG-APT4PP was determined to be 50 ± 6 mg of Pb per gram. The material demonstrates a strong affinity for lead, comparable to the performance of silica-based material functionalized with phosphonate groups [22]. Although this functionalized material does not compete with several materials already reported with exceptional performance in lead adsorption [9,13,14,15,16], the information obtained through this adsorption test provides novel insights at the molecular level to determine how lead ions interact with available phosphines. On the other hand, these findings highlight the effectiveness of incorporating phosphorus-containing functional groups as a strategic approach for designing advanced materials for selective interactions, particularly with heavy metals.

### 2.2. Surface Textural Properties

**Figure 1:**
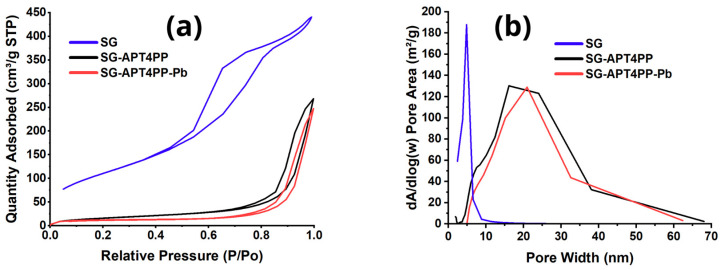
(**a**) Nitrogen adsorption–desorption isotherms, and (**b**) BJH pore size distribution of SG-APT4PP (black line) and SG-APT4PP-Pb (red line).

Textural characterization of SG, SG-APT4PP, and SG-APT4PP-Pb materials through nitrogen adsorption–desorption isotherms revealed significant structural changes resulting from Pb^2+^ ion interactions (Figure 1 and Table 1). The resulted isotherms correspond to type IV according to the IUPAC classification, typically associated with mesoporous materials [23]. A distinct hysteresis loop (type H3) was noted, suggesting the presence of mesopores with slit-like or plate-like shapes formed by the aggregation of particles [23,24,25]. The non-functionalized silica gel (SG) exhibited a high BET surface area of 406.09 m^2^/g, a total pore volume of 0.70 cm^3^/g, and an average pore diameter of 5.22 nm, confirming its mesoporous nature with a significant fraction of small mesopores. Upon functionalization with APT4PP, a dramatic reduction in surface area was observed from 406.09 m^2^/g to 21.12 m^2^/g, indicating extensive coverage of the internal surface by the organic moieties. This modification also led to a notable decrease in pore volume (from 0.70 to 0.41 cm^3^/g), accompanied by a substantial increase in average pore diameter (from 5.2 to 19.74 nm), suggesting partial pore blocking and preferential suppression of smaller mesopores during the grafting process. A notable decrease in BET specific surface area was evidenced from 21.12 m^2^/g for SG-APT4PP to 12.78 m^2^/g for SG-APT4PP-Pb, clearly demonstrating effective lead adsorption within the porous structure. This significant reduction can be directly attributed to the occupation of the available active adsorption sites by Pb^2+^ ions, effectively blocking nitrogen accessibility to internal surfaces and consequently limiting the available surface area for adsorption. Additionally, pore volume (Vp) showed a moderate decrease from 0.41 cm^3^/g to 0.38 cm^3^/g after lead adsorption, reinforcing the hypothesis that adsorption primarily occurs on readily accessible regions rather than completely blocking internal pores. Such a slight reduction in pore volume suggests that lead ions predominantly interact with the pore entrances and external surface areas, causing partial occupancy rather than complete occlusion of internal porous channels. Moreover, an increase in the average pore diameter (Dp) from 19.74 nm to 21.66 nm post-adsorption was observed. This shift in pore size distribution implies selective occupancy and saturation of smaller mesopores by Pb^2+^ ions, leaving a relatively higher proportion of larger mesopores available. Such a redistribution provides additional confirmation of the preferential adsorption mechanism taking place in smaller and more accessible pores. The pore size distribution analysis (Figure 1a,b) corroborates this interpretation by highlighting specific changes within the 5–40 nm pore range, clearly affected by Pb^2+^ ion interactions. The comparison with the non-functionalized silica gel (SG) further confirms the transformation of the textural properties across the functionalization and adsorption steps. The disappearance of the intense peak centered at ~5 nm (Figure 1b) in the SG sample and the emergence of a broader distribution centered around 20 nm in the modified materials underscore the structural reorganization and surface tailoring achieved by APT4PP grafting. Collectively, these structural analyses offer comprehensive views into the adsorption mechanism of Pb^2+^ onto SG-APT4PP, affirming its potential efficacy for selective heavy metal ion removal applications of this functionalized silica gel adsorbent.

### 2.3. FTIR Analysis

**Figure 2:**
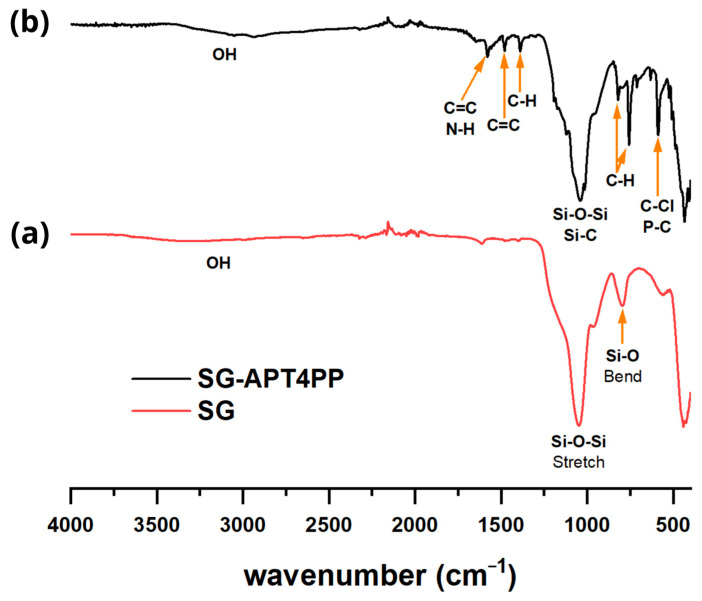
FTIR spectra: (**a**) silica gel (SG) and (**b**) SG-APT4PP.

Figure 2 shows the spectrum of the non-functionalized silica gel (SG) material, featuring two characteristic bands of SiO_2_ at 1045 cm^−1^ and 800 cm^−1^, attributed to Si-O-Si stretching and Si-O bending vibrations, respectively. In the case of the spectrum of functionalized silica gel (SG-APT4PP) reveals new signals confirming the presence of the APT4PP ligand. These include the N-H bending vibration at 1581 cm^−1^ of the amino group, the C=C stretching at 1476 cm^−1^ from phenyl groups, aliphatic C-H bending vibration at 1388 cm^−1^ from the propyl chain, as well as the aromatic ring bending at 816 cm^−1^ and 752 cm^−1^. Finally, the signal at 588 cm^−1^ is attributed to the stretching vibration of C-Cl and P-C bonds [26,27].

### 2.4. NMR Analysis

**Figure 3:**
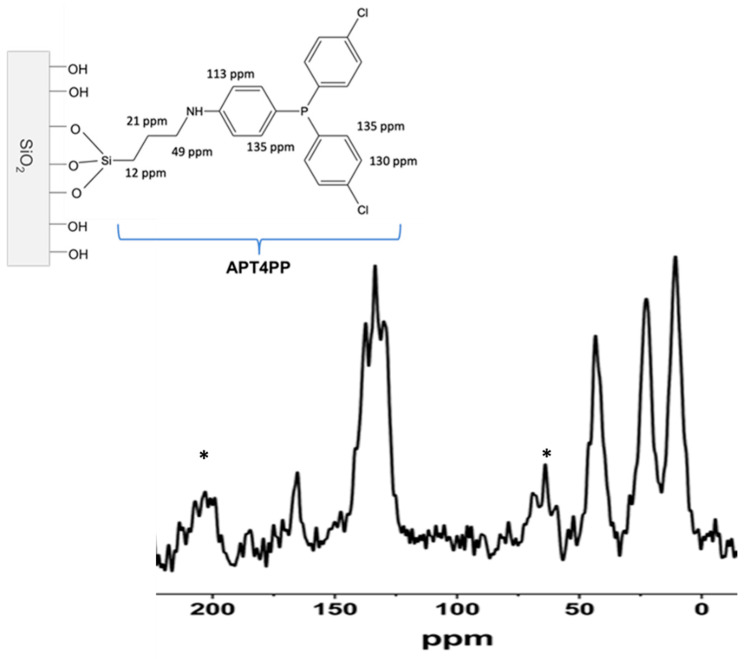
^13^C CP-MAS NMR spectrum of SG-APT4PP and its carbon chemical shift assignment. Spinning side bands of the aromatic signals (*).

Figure 3 shows the ^13^C CP-MAS NMR spectrum of SG-APT4PP. The three signals at 12, 21, and 43 ppm are attributed to the propyl carbon binding chain, and the broad signal, split in three on the top, between 125 and 135 ppm, belongs to all aromatic carbon signals of the T4PP head, confirming the proposed structure [28]. The signal at 165 ppm is possibly attributed to the formation of carbamate from unreacted amino propyl residue (AP) and atmospheric CO_2_, as was previously reported by Schimming et al. (1999) [29]. Signals with asterisks are due to the spinning side bands of the aromatic carbon signal.

**Figure 4:**
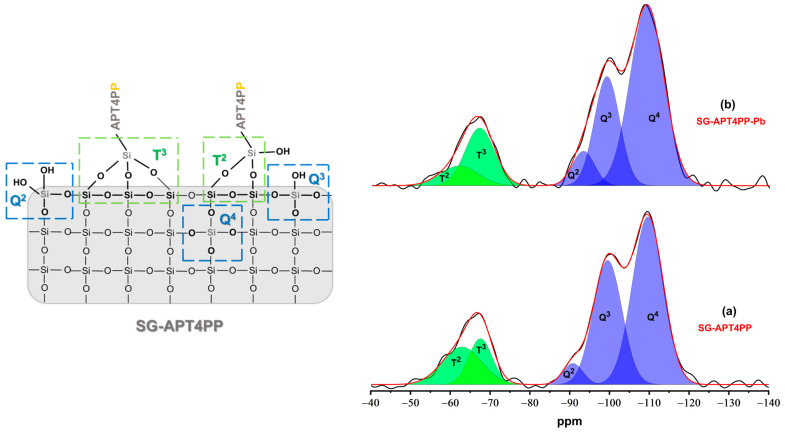
^29^Si MAS NMR spectra of (**a**) SG-APT4PP and (**b**) SG-APT4PP-Pb adsorbents.

The ^29^Si MAS NMR spectra (Figure 4a,b) provide valuable insights into the silicon environments present in SG-APT4PP and SG-APT4PP-Pb adsorbents. Both samples exhibit characteristic signals corresponding to Q– and T–silicon species, confirming the formation of the SiO_2_ matrix and successful surface functionalization. These signals remain largely unchanged after Pb^2+^ adsorption, suggesting that the functional groups are preserved.

In the SG-APT4PP sample, distinct silicon signals related to the silica gel matrix (Q) and the organic ligand (T) environments are observed at approximately −63.0 ppm (T^2^, Si (OSi)_2_ (OH)R), −67.6 ppm (T^3^, Si (OSi)_3_R), −90.8 ppm (Q^2^, Si (OSi)_2_ (OH)_2_), −99.5 ppm (Q^3^, Si (OSi)_3_OH), and −109.6 ppm (Q^4^, Si (OSi)_4_) [30,31,32]. Following Pb^2+^ adsorption (SG-APT4PP-Pb), these signals exhibit minor shifts and variations in intensity (Table 2). Given the amorphous nature of the material, the observed NMR signals are broad and cannot be narrowed by spectral processing techniques. This results in considerable overlap of the peaks, making it difficult to precisely determine their positions and intensities. Consequently, it remains unclear whether the subtle spectral differences between the two samples reflect genuine surface chemical changes due to Pb^2+^ adsorption or whether they are artifacts arising from the mathematical deconvolution of the broad, overlapping signals.

### 2.5. XPS Analysis Results

**Figure 5:**
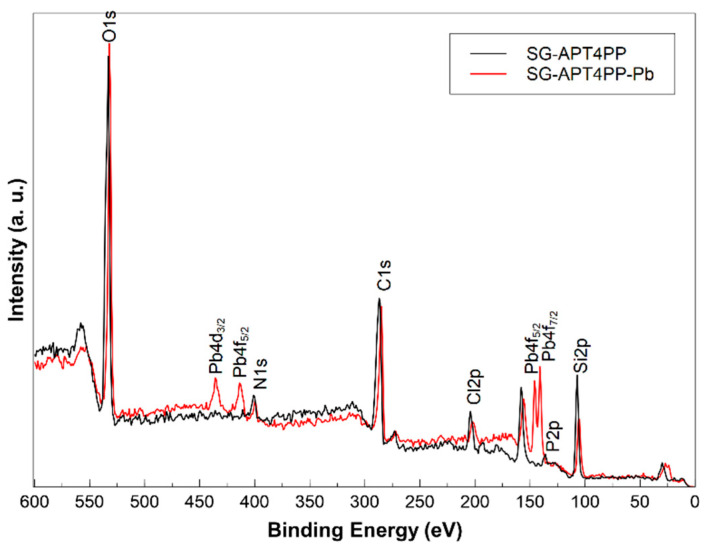
Wide-scan XPS spectra of SG-APT4PP and SG-APT4PP-Pb.

X-ray photoelectron spectroscopy (XPS) analysis was performed on the SG-APT4PP and SG-APT4PP-Pb samples to investigate the surface chemical composition and the interaction between phosphorus (P) and lead (Pb) (Figure 5). In both samples, the signals correspond to C1s, O1s, N1s, Cl2p, Si2p, and P2p. In the SG-APT4PP-Pb sample, clear signals corresponding to both P and Pb were detected, confirming metal adsorption. The Pb 4f core-level spectra revealed characteristic peaks corresponding to Pb (II) species, while the P 2p region showed the presence of phosphine groups [22,33].

**Figure 6:**
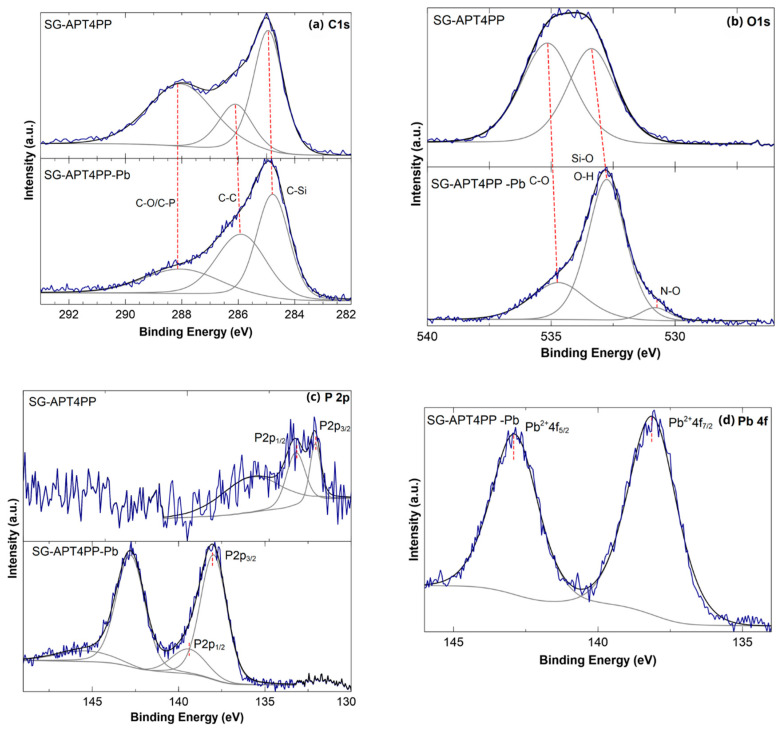
XPS spectra of SG-APT4PP and SG-APT4PP -Pb samples: (**a**) C 1s, (**b**) O 1s, (**c**) P 2p, and (**d**) Pb 4f.

The C 1s spectrum of SG-APT4PP (Figure 6a, top) was deconvoluted into three main components ranged at approximately 284.6, 285.5, and 286.4 eV, which were attributed to C–Si, C–C, and C–O/C–P bonds, respectively. These signals are consistent with the expected chemical structure of the silane-grafted phosphine ligand [22,34]. After Pb (II) adsorption, the C 1s spectrum of SG-APT4PP-Pb (Figure 6a, bottom) maintained the same three components with minor shifts of binding energy, indicating that the ligand functional groups remain chemically stable after metal uptake. The O 1s spectrum of SG-APT4PP (Figure 6b, top) was fitted with two primary peaks located at approximately 533.38 eV and 535.15 eV, assigned to O-H/Si–O and C–O bonds, respectively [33,35]. These components indicate the presence of oxygen atoms involved in both siloxane and organic functionalities, such as OH groups from silanols (Si-OH), bridging oxygens in siloxane bonds (Si-O-Si), and C-O bonds from remaining methoxysilane groups (Si-O-CH_3_). Upon interaction with Pb (II), the O1s spectrum of SG-APT4PP-Pb (Figure 6b, bottom) revealed three resolved components, indicating modifications in the local oxygen environment. A new peak at 530.81 EV was assigned to N-O interactions from nitrate anions (NO_3_^−^) present in the Pb (NO_3_)_2_ salt. This suggests that lead coordinates with oxygen atoms [36,37,38], supporting the proposed interaction mechanism, in which the phosphorus atom from the phosphine-type group participates in the stabilization process.

The high-resolution XPS spectrum in the P 2p region (Figure 6c) reveals important changes in the chemical environment of phosphorus upon Pb (II) coordination. In the SG-APT4PP spectrum, the P 2p signal (Figure 6c, top) shows two principal peaks attributed to P 2p_3/2_ and P 2p_1/2_ components of phosphine-type groups at 132.19 eV and 133.38 eV, respectively. After the interaction with Pb^2+^, the SG-APT4PP-Pb spectrum (Figure 6c, bottom) shows a shift to 138.70 eV (P 2p_3/2_) and 139.83 eV (P 2p_1/2_). The binding energy of P 2p increased by approximately 6.5 eV, indicating that the phosphorous atoms donate electron density to the lead ions during coordination, resulting in a decrease in electron density around phosphorous [39].

The Pb 4f spectrum of SG-APT4PP-Pb (Figure 6d) shows two well-defined peaks at 138.11 eV and 142.87 eV, corresponding to Pb 4f_7/2_ and Pb 4f_5/2_, respectively. A binding energy shift of 1.4 eV is observed, which can be ascribed to the coordination of phosphorus groups with Pb^2+^. This suggests that electron density is transferred from phosphorus to lead ions during the adsorption process [40,41]. The clear separation and intensity of the Pb 4f components support the successful interaction between Pb^2+^ ions and electron-donating groups, such as phosphine moieties, present in the material. These results are consistent with the shift observed in the P 2p region (Figure 6c) and collectively confirm the coordination of Pb^2+^ through electron donation from phosphorus-containing groups [21,42].

### 2.6. Evaluation of Lead Interaction by ^31^P NMR

**Figure 7:**
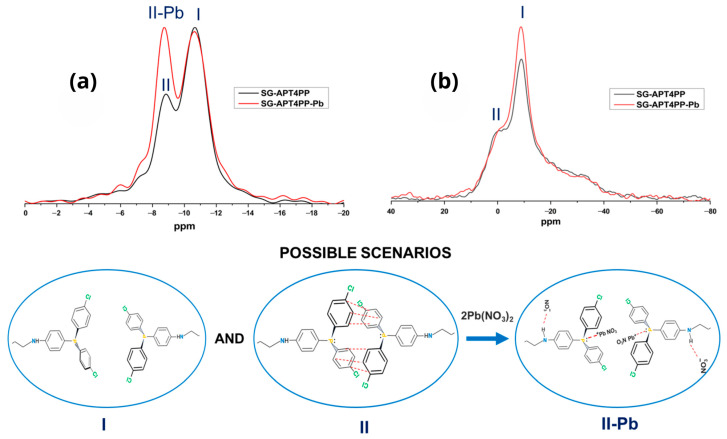
(**a**) MAS and (**b**) static ^31^P{^1^H} CP NMR spectra of SG-APT4PP (black) and SG-APT4PP-Pb (red).

Qualitatively, both the MAS and static ^31^P NMR spectra of SG-APT4PP appear similar before and after lead loading. The MAS spectra show two peaks at −8.8 (II) and −10.7 ppm (I), Figure 7a. The static spectra exhibit a single anisotropic signal, Figure 7b. Line-shape analysis of this static signal shows that it corresponds to the second peak in the MAS spectra, while the −8.8 ppm peak remains isotropic. The only change caused by lead loading is an increase in the relative intensity of the −8.8 ppm (II-Pb) peak compared to the −10.7 ppm peak, shifting the ratio from 3:10 to 7:10.

The most plausible explanation for these findings is the presence of two types of surface phosphine groups. One type exhibits slow rotational diffusion, characterized by an isotropic chemical shift of −10.7 ppm, a span (Ω) of 42 ppm, and a skew (κ) of 0.8. These values are consistent with those of phosphines in the crystalline state [43]. The other type undergoes fast rotational diffusion, which averages out the anisotropy of its CST, yielding an isotropic peak at −8.8 ppm. Similar mobility-driven transitions, induced by thermal or hydration effects on branched organic molecules in sub-monolayer amounts loaded onto mesoporous silica, have been previously reported [44]. The chemical shift difference between these signals cannot be attributed to interactions with lead cations or conversion to phosphine oxide; instead, it reflects variations in the ordering of phenyl rings in the two structures.

The interaction between these functional groups and lead cations is weak, resulting in no measurable changes in chemical shifts. This suggests that the adsorbent can be regenerated. Note that when phosphorus is coordinated to a transition metal center, its isotropic ^31^P chemical shift can change by more than 100 ppm [45]. However, lead loading increases the number of rotationally mobile functional groups (II-Pb). This implies that the mechanism restricting their rotational diffusion involves mutual interactions among these groups, which are disrupted in the presence of lead cations. This statement could also support the explanation of the increased pore size in the textural surface analysis of the material after lead absorption (SG-APT4PP-Pb). On the other hand, enhancing the interaction with lead cations can be achieved by converting the phosphine groups into phosphine oxide groups [22].

Furthermore, these observations are consistent with the findings obtained by XPS measurements, where changes in binding energies of P from phosphine groups were observed upon Pb^2+^ binding, supporting the presence of specific P--Pb interactions at the atomic level.

In summary, the present work demonstrated the possibility of investigating the lead uptake affinity, at the molecular level, of the phosphorus functionalized silica gel adsorbents by the direct evaluation of the Pb–P interaction through ^31^P NMR spectroscopy and corroborated by XPS analysis. Furthermore, this molecular approach represents valuable information for the design of more efficient functionalized adsorbents involving analogous and cost-effective materials.

## 3. Conclusions

A novel functionalized silica gel adsorbent, SG-APT4PP, was successfully synthesized using a one-pot sol–gel reaction through TEOS, APTES, and T4ClPP precursors. Spectroscopic characterizations, including FTIR, ^13^C and ^29^Si solid-state NMR, confirmed the presence of active organic phosphine grafted onto the silica gel matrix in the form of T^2^ and T^3^ species, as well as the tridimensional silica gel matrix by Q^2^–Q^4^ structures.

Lead removal tests revealed adsorption capacity of up to 50 mg g^−1^, confirming the specific adsorptive affinity of this material toward lead cations. Furthermore, lead presence is also observed in the reduction in the specific surface area of the material up to 60%. ^31^P NMR CST analysis identified two distinct types of surface phosphine groups exhibiting different rotational diffusion behaviors. For one type, the characteristic rotational reorientation time is much longer than milliseconds (slow diffusion), while for the other, it is significantly shorter (fast diffusion). Although the isotropic chemical shift values of these signals remained unchanged in the presence of lead cations, noncovalent interactions between neighboring phosphine groups are disrupted, leading to a marked increase in the number of groups with high rotational mobility. Furthermore, the P–Pb interaction was corroborated by XPS analysis through changes in the binding energies of the phosphine moieties upon lead uptake.

Finally, while the scope of this work was not related to reporting a competitive functionalized silica gel material for lead sorption, the novel contribution of one-pot synthesis and the molecular-level understanding of the interactions between phosphorus moieties and lead cations highlight the potential of this research to design new materials with selective sorption of heavy metals for environmental applications.

## 4. Materials and Methods

### 4.1. Materials

Tetraethyl orthosilicate (TEOS, ≥99%), 3-aminopropyltrimethoxysilane (APTMS) (≥97%), Tris (4-chlorophenyl) phosphine (T4ClPP) (≥95%), triethylamine (TEA, ≥99%), lead nitrate (99.9%), and nitric acid (Ultrex) were purchased from Sigma-Aldrich (Toluca, Mexico). Absolute ethanol and sodium chloride (≥99%) were supplied by Fermont (Monterrey, Mexico). All the reactants were used as received.

### 4.2. Synthesis of SG-APT4PP

The synthesis route used for SG-APT4PP was adapted from sol–gel methodologies previously developed and optimized by our group for the functionalization of mesoporous silica with organophosphorus ligands [22,46,47]. The experimental conditions were selected to ensure efficient incorporation of the tris (4-chlorophenyl)phosphine moiety into the silica matrix while maintaining structural homogeneity and stability of the hybrid network.

**Figure 8:**
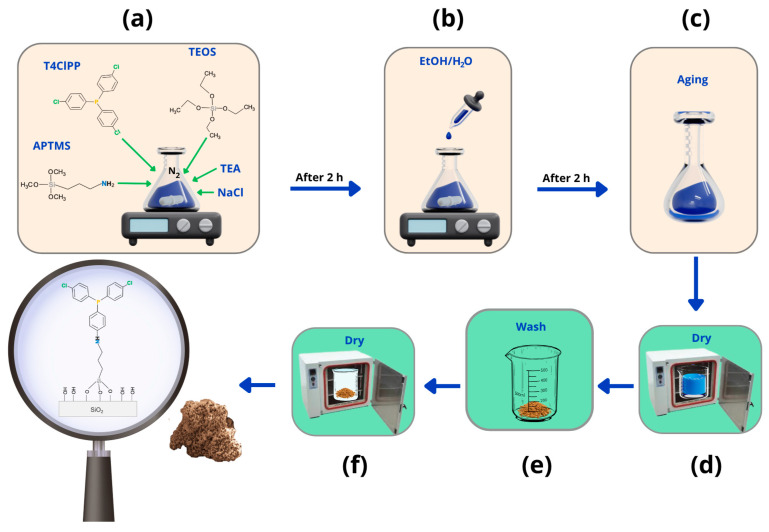
One-pot synthesis (**a**–**c**) and the cleaning steps (**d**–**f**) of SG-APT4PP.

The synthesis of SG-APT4PP was carried out according to the sol–gel procedure illustrated in Figure 8a–f. Initially (Figure 1a), in a one-pot reaction, a mixture of reactants tetraethyl orthosilicate (TEOS, 10 mM), Tris (4-chlorophenyl) phosphine (T4ClPP, 1.1 mM), and (3-aminopropyl) trimethoxysilane (APTMS, 3.3 mM) was prepared in the presence of sodium chloride (NaCl, 1 mM) under nitrogen atmosphere. Triethylamine (TEA, 0.3 mL) was added dropwise as a base catalyst, and the reaction mixture was stirred for 2 h at room temperature to promote precursor hydrolysis and co-condensation. Subsequently, 4.4 mL of a solution of ethanol and deionized water (0.8% *v*/*v*) was added to the mixture and stirred for 2 h to ensure the formation of silanol groups and their condensation (Figure 8b). The resulting material was aged for 2 days at room temperature to allow the polymeric network development (Figure 8c). After aging, the gel material was dried at 60 °C for 24 h to remove residual solvents (Figure 8d). The xerogel was then washed thrice using a 1:1 (*v*/*v*) ethanol/deionized water mixture to eliminate unreacted precursors and byproducts (Figure 8e), followed by a final drying step at 60 °C for 24 h (Figure 8f). The xerogel corresponds to the hybrid silica gel material functionalized with APTMS and T4ClPP was named SG-APT4PP.

### 4.3. SG-APT4PP Characterization

The specific surface area (BET) was determined from nitrogen adsorption–desorption isotherms at 77 K using an ASAP 2020 KMP analyzer (Micromeritics, Norcross, GA, USA) in the relative pressure range of 0.05 < P/P_0_ < 0.35. The analysis was conducted on SG-APT4PP before and after the lead adsorption test. The chemical composition of SG-APT4PP and a control of non-functionalized silica gel (SG) was evaluated by FTIR spectroscopy using a Thermo Fisher Scientific Nicolet iS50 Model equipment, with an ATR device. Samples were acquired with 16 scans using a spectral width range of 500–4000 cm^−1^ and a resolution of 4 cm^−1^.

Solid-state NMR measurements of SG-APT4PP samples were performed at ambient temperature on an Infinityplus NMR spectrometer system (Agilent) operated at 7 T (300 MHz), equipped with a 6 mm CP-MAS probe of variable-temperature Chemagnetics-Varian. Samples were packed into 6 mm pencil rotor of ZrO_2_ and measured with a spinning speed of 7 kHz, with a 90° pulse length of 4.0 μs, a relaxation delay of 3 s. Contact times were set to 3 ms, 10 ms, and 1 ms for ^13^C {^1^H}, ^29^Si{^1^H}, and ^31^P{^1^H} CP NMR, respectively. Further experimental details can be found in [44].

Elemental composition and chemical states of the functionalized silica gel adsorbent before and after lead uptake were studied by a SPECs Flex SP X-Ray Photometry (XPS) using a monochromatic Al Kα source (hv = 1486.7 eV) and a pass energy of 10 eV. Spectra were analyzed using CasaXPS software version 2.3.24.

### 4.4. Lead Removal Test

Since the scope of this research is oriented to the evaluation of lead–phosphines interaction, neither equilibrium adsorption models nor kinetics were performed. However, in a triplicate experiment, the maximum Pb uptake was measured to evaluate the interaction. Functionalized silica gel adsorbent (SG-APT4PP) was treated with a 3000 mg L^−1^ stock solution of Pb at pH 3, prepared using lead nitrate and adjusting the pH with 1% HNO_3_. A total of 100 mg of the adsorbent was placed in a flask containing 25 mL of the lead solution. The flasks were stirred for 24 h on a thermostirrer (Thermo Fisher Scientific, Cincinnati, OH, USA) set to 120 rpm at 303.15 K. Afterward, the solution was filtered, and the initial and final Pb concentrations were measured by an Agilent 240FSAA Atomic Absorption Spectrometer (Agilent Technology, Santa Clara, CA, USA).

Calibration curves were used to construct concentration vs. absorbance using a certified standard of Pb ion solution (Sigma-Aldrich, St. Louis, MO, USA). The lead adsorbed amount on the SG-APT4PP adsorbent was obtained between the initial and final lead concentration (Equation (1)):

(1) $$q=\frac{V\left(C_{0}-C_{f}\right)}{m}$$

where *q* is the amount of lead adsorbed (in mg g^−1^); *C*_0_ and *C_f_* are lead concentrations (in g L^−1^) in the initial and final concentrations, respectively; *V* is the volume of solution with lead ions (L); and *m* is the amount of adsorbent (g).

## Figures and Tables

**Table 1:** Structural parameters of adsorbents (V_p_ = pore volume, D_p_ = mean pore diameter).

Sample	BET Surface Area	Vp	Dp
	SBET (m^2^g^−1^)	cm^3^g^−1^	nm
**SG**	406.09	0.70	5.22
**SG-APT4PP**	21.12	0.41	19.74
**SG-APT4PP-Pb**	12.78	0.38	21.66

**Table 2:** ^29^Si CP-MAS NMR chemical shifts for different silicon species in SG-APT4PP and SG-APT4PP-Pb samples.

Sample	Chemical Shift (ppm)					Q/T
	T^2^	T^3^	Q^2^	Q^3^	Q^4^	
SG-APT4PP	−63.0 (2.7, 13%) ^a^	−67.6 (1.9, 9%) ^a^	−90.8 (0.8, 4%) ^a^	−99.5 (6.4, 30%) ^a^	−109.6 (9.4, 44%) ^a^	3.6
SG-APT4PP-Pb	−61.9 (1.2, 6%) ^a^	−67.1 (2.8, 14%) ^a^	−93.2 (1.3, 6%) ^a^	−99.1 (4.5, 23%) ^a^	−109.1 (10.1, 51%) ^a^	3.9

^a^ Peak areas and percentages are provided in parentheses.

## Data Availability

The original contributions presented in this study are included in the article. Further inquiries can be directed to the corresponding author.
